# Systemic Sarcoidosis Presenting With Hypercalcemia, Acute Kidney Injury, and Diffuse Lymphadenopathy: Unlocking Pandora’s Box

**DOI:** 10.7759/cureus.85882

**Published:** 2025-06-12

**Authors:** Virginia Geladari, Paraskevi Liaveri, Georgios Liapis, Georgios Moustakas, Nikolaos Sabanis

**Affiliations:** 1 1st Department of Internal Medicine, General Hospital of Trikala, Trikala, GRC; 2 Department of Nephrology, General Hospital of Athens “Georgios Gennimatas”, Athens, GRC; 3 1st Department of Pathology, National and Kapodistrian University of Athens, Athens, GRC; 4 Department of Nephrology, General Hospital of Trikala, Trikala, GRC

**Keywords:** acute kidney injury, granulomatous interstitial nephritis, hypercalcemia, renal sarcoidosis, sarcoidosis

## Abstract

Sarcoidosis is a rare granulomatous disease with complex pathogenesis, nonspecific manifestations, and systemic sequelae leading to difficulties in differential diagnosis and increased potential for misdiagnosis. Renal involvement consists of an uncommon and underreported condition, especially in the absence of other extra-renal manifestations.

Herein, we describe the case of a 58-year-old Caucasian female who presented with acute kidney injury alongside persistent hypercalcemia, diffuse lymphadenopathy, and B symptoms, namely, unintentional weight loss, fever, and fatigue. The laboratory findings revealed elevated creatinine and calcium levels, suppressed intact parathyroid hormone (iPTH) and increased calcitriol and angiotensin-converting enzyme (ACE) levels, raising suspicion of sarcoidosis. Renal biopsy revealed the presence of non-necrotizing granulomas, a pattern compatible with the diagnosis of sarcoidosis granulomatous interstitial nephritis (sGIN). Combined therapy with corticosteroids and hydroxychloroquine was initiated, and the patient’s follow-up showed significant improvement in kidney function without relapses, highlighting the importance of early disease recognition and intervention.

This case study unveils the diagnostic odyssey of the clinician to establish the diagnosis of sarcoidosis and prompts them to include such a diagnosis in their differential diagnosis algorithm in patients presenting with acute kidney injury, diffuse lymphadenopathy, and non-PTH-mediated hypercalcemia.

## Introduction

Sarcoidosis is considered an idiopathic inflammatory granulomatous disease characterized by the formation and accumulation of non-necrotizing granulomas in almost every organ. The first disease reference was made in 1878 by the physician Dr. Hutchinson, while in 1889 Dr. Besnier first described lupus pernio and some years later, in 1899, Dr. Boeck isolated and named the first sarcoid granuloma from skin biopsy samples [1].

Although the discovery of sarcoidosis dates back more than 150 years, its underlying etiology and origin remain elusive. Nowadays, the pathogenesis of sarcoidosis is mainly attributed to the complex interplay between several environmental stimuli and genetic predisposition [2-3]. The global distribution and incidence of sarcoidosis vary between different geographical regions, races, ethnicities, and it is inextricably associated with the available diagnostic resources, technologies, and level of health care experience. The disease incidence is estimated between 1 and 15 cases per 100,000, and it is higher in Scandinavians, Northern Europeans, and African Americans and lower in Asian populations. Both sexes are equally affected, and epidemiological data suggest a bimodal distribution regarding age; an early appearance in ages under 40 years mainly with male predominance and a second age peak between 50-60 years with primarily female superiority [3-7].

Sarcoidosis is characterized as a “kaleidoscopic disease” due to its great heterogeneity in clinical symptomatology that mimics several other conditions. Clinical manifestations, organ involvement, severity, and prognosis range widely depending on age, gender, and race, making this protean disease diagnosis, a diagnosis of exclusion. Although intrathoracic involvement remains the rule in the disease course, presenting in 90% of cases, almost every extrapulmonary organ has been reported to be affected, namely skin, eyes, joints, liver, spleen, nervous system, heart, and kidneys [1,3,8].

Herein, this case describes a 58-year-old female with sarcoidosis, primarily presenting with renal manifestations due to multiple underlying sarcoidosis-related conditions. We also briefly review the available literature on renal involvement in sarcoidosis and detail the diagnostic journey leading to a definitive diagnosis through a kidney biopsy, which revealed the presence of sarcoidosis granulomatous interstitial nephritis (sGIN), a rather uncommon and usually random histopathological pattern of renal injury, despite autopsy studies estimating its prevalence at approximately 7-23% of cases [9].

## Case presentation

A 58-year-old Caucasian woman presented at the Emergency Department, reporting drowsiness and fatigue along with the presence of polyuria, polydipsia, and low-grade fever during the last few days. She also mentioned unintentional weight loss; approximately 20 kilograms over the past four months. Additionally, she denied recent medication changes, vaccinations, or infections. Her past medical history was unremarkable apart from arterial hypertension and gastroesophageal reflux disease. The patient’s daily medication included amlodipine 5 mg once daily (o.d.), nebivolol 2.5 mg o.d., and esomeprazole 40 mg o.d.

On arrival, her vital signs included blood pressure of 130/75 mmHg, heart rate of 90 bpm, oxygen saturation of 95% on room air, and a low-grade fever of 37.4°C. Although the patient complained about somnolence and decreased level of consciousness, no neurological deficit was observed. Overall clinical examination was also unremarkable, apart from the presence of swollen, palpable, and painless bilateral axillary, cervical, and supraclavicular lymph nodes.

Initial laboratory examination revealed acute kidney injury (AKI) with serum creatinine (sCre) levels of 4.7 mg/dL, serum urea (sUrea) levels of 85 mg/dL, and severe hypercalcemia with serum calcium levels of 14.4 mg/dL. Urinalysis was positive for hemoglobin (++) and a moderate amount of protein (++), while urine sediment showed the presence of 60-80 leukocytes and 10-20 erythrocytes per high-power field (hpf). Furthermore, urine microscopy did not detect the presence of dysmorphic erythrocytes or urine casts, such as fatty casts, granular, renal tubular epithelial cells, or white blood cell casts.

Kidney and neck ultrasound imaging were also performed. The findings showed kidney size and renal cortex echogenicity within normal range without evidence of nephrolithiasis or obstruction, while cervical ultrasound examination demonstrated the presence of an enlarged right lobe thyroid bronchocele, together with the presence of a benign left lobe nodule and multiple swollen anterior and posterior cervical lymph nodes.

Due to the above findings, the patient’s admission was decided for further investigation and treatment. Intravenous infusion of isotonic normal saline solutions was initially administered due to volume depletion and under a point-of-care ultrasound because of possible fluid intolerance in the context of AKI. After restoring intravascular volume, the patient’s calcium levels remained significantly elevated. Thus, furosemide and intravenous administration of bisphosphonate (2 mg of zolendronic acid) were administered while a urine catheter had been placed in order to record patients’ daily urine excretion.

Further laboratory testing in order to determine the cause of patient’s hypercalcemia showed severely decreased iPTH levels (4.8 pg/mL; normal range: 17-88 pg/mL) together with increased 1,25-dixydroxy vitamin D (1,25(OH)2 vitamin D) levels (90 ng/mL; normal range: 30-50 ng/mL). We initially suspected the possibility of vitamin D3 intoxication; however, the patient denied any external vitamin supplement use. The 24-hour urine collection showed protein excretion at 1.4 g/24 h as well as hypercalciuria with urine calcium excretion at 320 mg/24 h.

Interestingly, the patient’s hypercalcemia was non-PTH related, and therefore our differential diagnosis included underlying infectious diseases, malignancies, lymphomas, and granulomatous diseases. Taking into consideration the patient’s constitutional symptoms (weight loss, fatigue, and low-grade fever) combined with clinical and laboratory findings (acute renal failure, hypercalcemia, lymphadenopathy), a thorough investigation was scheduled.

Total body computed tomography (CT) did not demonstrate the presence of solid tumors, hepatomegaly, or splenomegaly; however, diffuse lymphadenopathy, mainly detected in the mediastinum, axillary, pro- and paratracheal, abdominal, and pro- and paraaortic sites, was noted. Simultaneously, upper and lower gastrointestinal endoscopy as well as digital breast tomosynthesis did not manage to demonstrate any pathological findings.

Complete screening for common viral and bacterial infections, such as hepatitis B virus, hepatitis C virus, HIV, cytomegalovirus, Epstein-Barr virus, *Toxoplasma*, *Leishmania*, *Leptospira*, and *Brucella*, came out negative. Consecutive urine and blood cultures were also sterile. Furthermore, targeted tuberculosis testing with interferon gamma release assay (IGRA-Quantiferon) recorded negative, excluding the above diagnosis.

Over the next days, the meticulous serum immunology testing results, including rheumatoid factor (RF), antinuclear antibodies (ANA), antineutrophil cytoplasmic antibodies (ANCA), anti-double stranded DNA (anti-dsDNA), complement 3 (C3), complement 4 (C4), cryoglobulins, total light κ- and λ- chains, serum and urine electrophoresis and immunofixation, were also negative. The only notable finding was the presence of increased serum angiotensin-converting enzyme (sACE) > 120 U/L (normal range: 8-52 U/L), compatible with the diagnosis of sarcoidosis.

Taking into consideration that sarcoidosis is a diagnosis of exclusion, additional investigation was arranged in order to exclude possible alternative causes of lymphadenopathy. A biopsy of the left axillary lymph nodes was carried out, showing findings compatible with reactive lymphadenitis, while a fine-needle aspiration (FNA) biopsy of the right lobe thyroid nodule ruled out the possibility of underlying malignancy. Simultaneously, in order to investigate the possibility of an underlying hematopoietic tumor, a bone marrow biopsy was scheduled. The morphological and immunohistochemical findings did not show infiltration by a lymphocytic population and excluded the diagnosis of lymphoid tissue malignancy.

**Figure 1:**
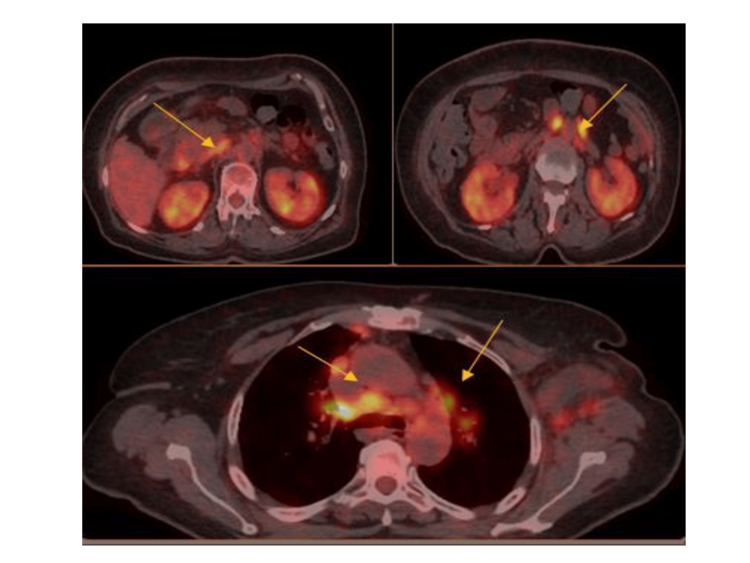
18-FDG PET/CT scan imaging 18-FDG PET/CT scan imaging revealed increased tracer uptake in the patient’s mediastinum, axillary, and abdominal lymph nodes. 18 FDG PET/CT: 18 F-fluorodeoxyglucose positron emission tomography

In addition, the patient underwent a fluorine 18 fluorodeoxyglucose (18-FDG) positron emission tomography (PET)/CT scan, which revealed the presence of increased tracer uptake in bilateral lymph nodes located in the upper and lower sites of the diaphragm, together with hypermetabolic nodules in the right upper lung lobe. The above findings, particularly increased tracer uptake in bilateral lymph nodes and hypermetabolic nodules in the right upper lung lobe, were highly relevant to assessing systemic involvement. Given sarcoidosis’s affinity for pulmonary structures, these imaging results aligned with expectations and further reinforced the diagnosis of sarcoidosis (Figure 1).

Due to the above findings, an endobronchial ultrasound (EBUS) followed by transbronchial lung biopsy, transbronchial needle aspiration, and bronchoalveolar lavage was arranged. Although biopsy results excluded the presence of malignancy, findings of cytology analyses were compatible with non-caseating granulomatous lymphadenitis, possibly but not definitely attributed to sarcoidosis.

Taking into consideration the patient’s sustained kidney impairment (sCr 2.5 mg/dL) despite supportive treatment with intravenous hydration and hypercalcemia correction, combined with high clinical suspicion of granulomatous interstitial nephritis (GIN) associated with sarcoidosis, we decided to perform a kidney biopsy. Meanwhile, immunosuppressant therapy was initiated with pulse steroid treatment (500 mg of intravenous methylprednisolone for three consecutive days) followed by oral methylprednisolone use at a dose of 40 mg per day. A few days later, the patient was transferred to a tertiary hospital where an ultrasound-guided left kidney biopsy was carried out.

**Figure 2:**
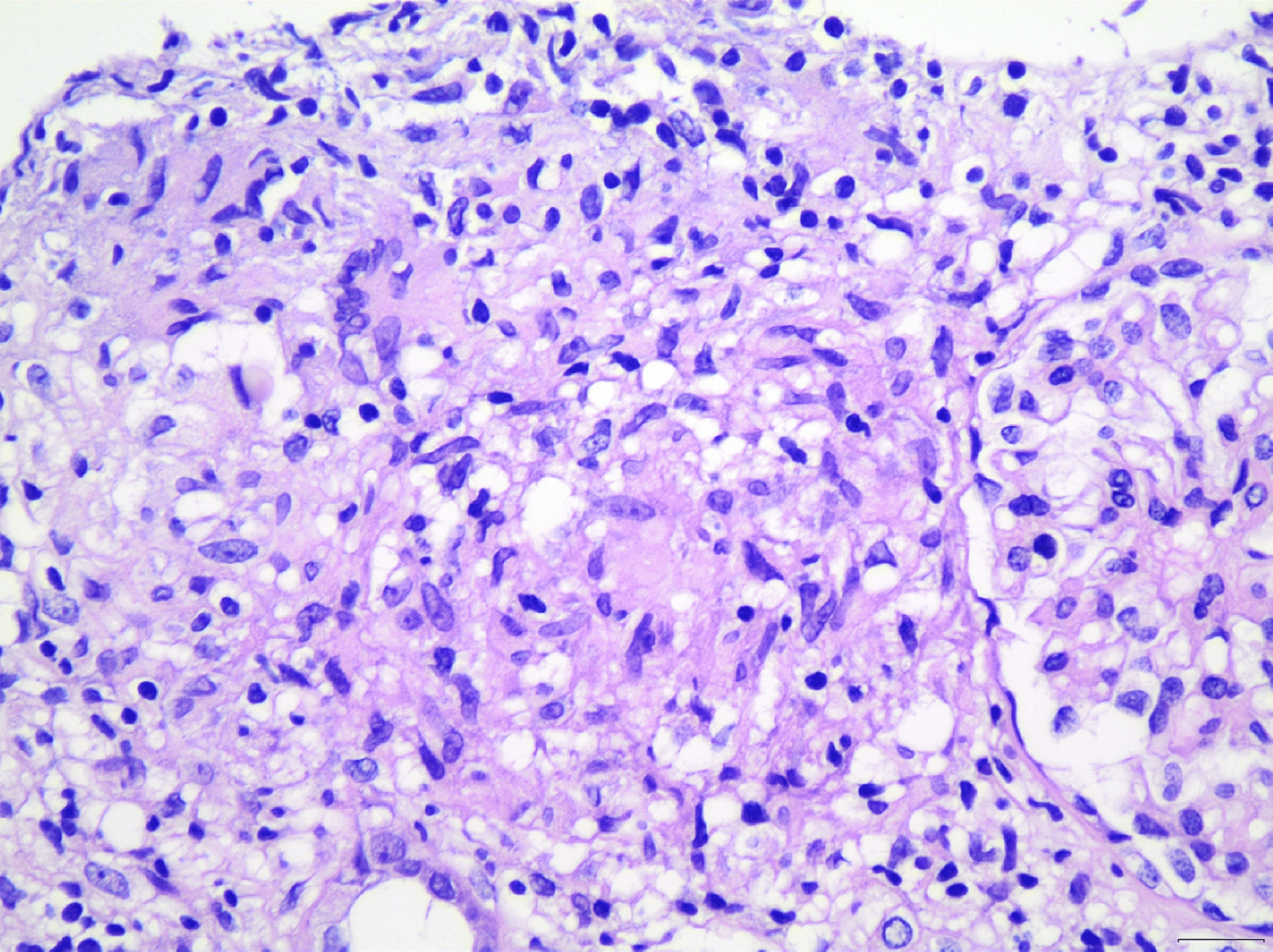
Kidney biopsy histopathology A well-formed granuloma, composed of epithelioid histiocytes, lymphocytes, and a giant cell in the center, can be seen adjacent to a glomerulus (H&E ×400).

Biopsy specimens consisted of two kidney cylinders; the first one, including seven glomeruli, was sent for light microscopy, while the second one, including five glomeruli, was sent for immunofluorescence. Light microscopy findings revealed slightly increased glomerular size, mesangial expansion, together with chronic inflammatory interstitial infiltrates consisting of lymphocytes, mast cells, eosinophils, and focal edema. Interestingly, a well-defined, non-necrotizing granuloma consisting of epithelioid mast cells, lymphocytes, and macrophages, as well as the presence of Langhans giant cells with multiple peripheral nuclei, was recognized in the renal cortex (Figure 2).

Although histopathological findings compatible with moderate interstitial fibrosis and tubular atrophy occupying 30-35% of substrate were also noted, these results were not considerably taken into account due to the small sample size. Additionally, coexistence of “hyaline droplets” with sites of acute tubular injury and microvacuolar tubular degeneration was observed. Congo-Red and Ziehl-Nielsen strains, together with immunohistochemical results for the detection of DNAJB9 marker, all revealed negative. Similarly, immunofluorescence was negative apart from the detection of immunoglobulin IgM (1+) and complement component C3 traces in the mesangium. Taken together, the above histological findings combined with the patient’s history advocated for GIN in the setting of renal sarcoidosis.

Thereafter, the patient continued oral methylprednisolone use in combination with hydroxychloroquine (200 mg/daily). One month after steroid treatment started, we also initiated dapagliflozin, a sodium-glucose cotransporter-2 inhibitor (SGLT-2i), at a dose of 10 mg o.d. In addition, we recommended avoiding sun exposure as well as exogenous administration of vitamin D supplements. Gradually, the patient’s kidney function and hypercalcemia improved (Table 1).

**Table 1:** The patient’s laboratory results throughout the 12-month follow-up. ESR: erythrocyte sedimentation rate; HbA1c%: concentration of glycated hemoglobin A1; TSH: thyroid-stimulating hormone

Variable	Day 1	Day 5	Day 10	Day 15	Day 20	Day 25	1 Month Later	2 Months Later	3 Months Later	6 Months Later	9 Months Later	12 Months Later	Normal Value or Range
White-cell count (10^3^/μL)	5.25	4.96	5.04	5.86	3.33	4.62	4.56	9.17	10.89	8.27	6.64	7.60	4-10.8
Hematocrit/Hemoglobin (%, g/dL)	38/13	36.4/12.8	34/12.1	36.4/12.7	33.3/11.5	33.3/11.7	33.0/11.3	36.3/12.6	37.7/13.3	35.2/12.1	38.9/13	37.5/12.6	11.8-17.8/37.7-47.9
Platelets (10^3^/μL)	307	223	221	260	182	204	246	280	289	289	241	228	150-350
Total proteins / Albumin (g/dL)	6.89/3.67	6.56/3/42	6.71/3.46	6.24/3.22	6.34/3.36	6.59/3.75	7.34/4.02	6.98/3.91	6.34/4.04	6.47/4.16	6.86/4.34	6.94/4.38	6.4-8.3/3.4-4.8
Glucose(mg/dL)	82	78	72	82	72	77	74	107	79	75	83	80	75-115
Urea (mg/dL)	85	75	69	76	60	66	60	128	96	68	72	58	10-50
Creatinine (mg/dL)	4.7	3.98	3.62	3.81	3.62	2.03	2.07	2.12	1.72	1.76	1.93	1.58	0.40-1.1
Potassium (mEq/L)	4.25	3.57	4.78	4.46	4.78	4.25	3.45	4.22	4.08	3.58	3.98	3.7	3.5-5.1
Sodium (mEq/L)	134.9	137.3	132.5	136.6	136.7	136.1	138.4	132.1	136.4	142.4	144.4	142.9	136-145
Calcium (mg/dL)	14.4	11.1	10.9	8.3	8.2	8.5	8	9.5	9.5	9.9	9.8	9.6	8.1-10.4
Phosphorus (mg/dL)	3.6	3.2	-	-	-	-	-	2.8	2.2	3.5	3.7	3.6	2.5-4.5
Magnesium (mg/dL)	1.41	1.26	2.1	2	-	-	-	1.75	1.5	-	1.8	-	1.6-2.3
Uric acid (mg/dL)	4.7	-	-	4.2	-	-	3.6	6.4	6.2	4.2	4.4	3.9	2.3-6.1
Intact-PTH (pg/mL)	4.7	4.8	-	-	-	-	-	67	65	70.6	82.3	60.5	17-88
ESR (mm/h)	24	-	29	-	25	-	-	-	23	14	-	26	0-15
HbA1c%	-	6	-	-	-	-	-	5.5	6.1	5.7	5.6	5.6	4-6%
TSH (mU/L)	-	2.19	-	-	-	-	-	1.78	1.57	1.1	2.05	1.76	0.5-4.5 mU/L
Urine volume	4700	4400	4250	-	-	-	-	2550	2500	3000	3000	2800	1000-2500 mL/24 h
Urine creatinine	-	-	-	-	-	-	-		52	-	930.3	-	14-26 mg/kg/24 h
Urine total protein	-	-	1400	-	-	-	-	341.7	345.8	477	425	309	<150 mg/24 h
Urine calcium	-	-	320	-	-	-	-	30.6	18.2	-	86.8	53.2	<250 mg/24 h
Urine sodium	-	-	-	-	-	-	-	51	39	69.5	116	-	50-200 mEq/24 h
Urine erythrocytes	60-80	-	0-2	0-2	-	-	2-4	1-3	0-2	0-2	0-2	0-2	3-5/hfp
Urine leukocytes	10-20	-	0-2	12-14	-	-	4-6	0-2	0-2	0-2	0-2	0-2	0-5/hfp

## Discussion

Sarcoidosis is an insidious disease characterized by most clinicians as the “great mimicker” due to its ability to manifest in various ways and resemble several other conditions, making the final diagnosis challenging. It is considered a rare, multisystem granulomatous disease, mainly distinguished by organ infiltration of non-caseating granulomas. Profound research has been done in order to define the underlying causes; however, the exact disease etiology remains unclear [1,3,7].

The interpretation behind granuloma formation in sarcoidosis is considered to be the result of a complex interaction between several environmental triggers, genetic susceptibility, and immune response. More specifically, the granuloma consists of a well-defined immune structure, which is actually the result of the unsuccessful elimination of an endogenous or exogenous pathogen. The granuloma nucleus includes both innate and adaptive immune cell infiltrations, mainly composed of macrophages and T-helper lymphocytes; however, accumulations of neutrophils, monocytes, and fibroblasts are also present. Various antigens have been incriminated as potential stimuli in sarcoidosis, such as viral and bacterial infections, including pathogens like mycobacteria and *Propionobacterium* acnes, organic and non-organic substances, as well as self-antigens like vimentin [10-13].

Despite environmental exposure, recent Genome-Wide Association Studies (GWAS) have identified a strong association with several predisposing genetic variants, including human leukocyte antigen (HLA) system genes, T-cell activation regulatory and cytokine genes, as well as pattern recognition receptor (PRR) encoding and autophagy loci. Indeed, new data suggest that some of these genetic polymorphisms are related to the disease severity. Positive family history also constitutes a vigorous risk factor for sarcoidosis appearance, increasing almost four times the possibility of manifesting the disease in first-degree relatives [10,13,14].

Recent reports suggest a rise in the recorded sarcoidosis prevalence, which ranges between 2.7 and 160 per 100,000 individuals and is most likely attributed to better diagnostic approach, technological tools, and higher clinical suspicion. Clinical presentation in sarcoidosis differs between individuals depending on gender, age, ethnicity, and regional criteria. Its course is usually unpredictable, appearing with a wide spectrum of more or less acute and significant symptoms [15-16].

Due to sarcoidosis complexity and the absence of a specific and sensitive genetic, clinical, laboratory, or radiological marker, the diagnosis is considered strenuous. In the vast majority, the disease diagnosis is confirmed after ruling out other inflammatory granulomatous conditions with similar clinical features, making sarcoidosis a diagnosis of exclusion. Actually, in order to establish sarcoidosis diagnosis, the following three criteria should be met: 1) Compatibility with the disease clinical presentation, 2) Isolation of non-caseating granulomas in at least one affected organ, and 3) Exclusion of any compatible alternative etiology [1,6,16].

Practically, almost every organ can be affected. However, the disease hallmark is considered to be the intrathoracic involvement with or without mediastinal lymphadenopathy, present in approximately 90% of cases. Extrapulmonary manifestations are heterogeneous and mainly include systemic lymphadenopathy, cutaneous and ocular sarcoidosis, musculoskeletal involvement, as well as hepatic and splenic manifestations, occurring in nearly one-fourth to one-half of cases. The presence of nonspecific symptoms such as fatigue, low-grade fever, sweats, consciousness disorders, and weight loss is also very common in sarcoidosis, affecting 50-70% of patients. On the other hand, central and peripheral nervous system, cardiac and kidney involvement are found in less than 10% of cases and remain quite rare [1,3,8,16].

In a clinical setting, renal sarcoidosis (RS) is an infrequent manifestation of the disease associated with increased mortality and morbidity. The exact prevalence of RS is quite difficult to determine since diagnostic criteria are not yet standardized, and most epidemiologic data come from small case series. The diagnosis of RS occurs most commonly between the fourth and fifth decades of life, and interestingly seems to have a reversed gender and race prevalence, with mainly male and white race predominance when compared to other most frequent sarcoidosis forms. In general, it is estimated that approximately one-third of sarcoidosis patients may manifest kidney involvement as a result of multiple different pathogenic mechanisms that occur in the disease course [17-21].

The spectrum of the underlying mechanisms implicated in RS pathogenesis is wide and can be divided in: 1) Calcium-associated abnormalities such as hypercalcemia, hypercalciuria, nephrolithiasis, nephrocalcinosis, and renal tubular acidosis, 2) Acute renal failure presenting as tubulointerstitial (granulomatous and non-granulomatous disease) or glomerular disease (minimal change disease, focal segmental glomerulonephritis, IgA nephropathy and membranous nephropathy), and 3) Obstructive uropathy due to retroperitoneal lymphadenopathy or fibrosis. The presence of kidney impairment in sarcoidosis is estimated at 0.7 to 4.3% of cases, and it is primarily attributed to calcium level disturbances leading to irreversible dialysis-dependent end-stage kidney damage in up to 10% of patients [17,22-23].

Indeed, patients with RS experience a diverse range of symptoms, with nearly 90% showing extra-renal involvement at the time of diagnosis. Isolated RS is extremely limited in the existing bibliography. Constitutional symptoms, including fatigue, sweats, weight loss, and low-grade fever, usually accompany patients with RS in almost one-half of the cases, and the above clinical picture was also noted in our patient. Usually, the aforementioned clinical scenario is the initial reason that leads patients to seek medical guidance [18-19].

Insufficient kidney function with abnormal urea and creatinine values and decreased estimated glomerular filtration rate (eGFR) are present in approximately 30-60% of diagnosed RS cases. Furthermore, urinary laboratory findings may include nonspecific findings like proteinuria, sterile pyuria, hematuria, glycosuria, and hypercalciuria. In fact, proteinuria characterizes approximately 65% of RS cases, and it is more frequently reported at sub-nephrotic levels [17-19].

As mentioned before, hypercalcemia and hypercalciuria are two of the most commonly seen manifestations of the disease. Hypercalcemia is present in approximately 35% of patients with RS and is both a result of sarcoidosis and a cause of worsening kidney function in the disease. Notably, elevated calcium levels in sarcoidosis are non-PTH-dependent, and the underlying pathogenetic mechanism is mainly correlated with granuloma-associated factors. More specifically, ectopic hyperproduction and expression of 1-alpha hydroxylase from activated macrophages in the sarcoid granuloma lead to overwhelming extra-renal conversion of 25-hydroxyvitamin D to 1,25-dihydroxyvitamin D, further leading to increased osteoclastic activity, elevated gastrointestinal absorption, and renal tubular reabsorption of calcium. In sarcoidosis, alternative hypercalcemia mechanisms suggest stimulation of 1-alpha hydroxylase by release of parathyroid hormone-related protein (PTH-rP) and increased excretion of interferon-γ. As a result, expression of parathyroid hormone (PTH) is suppressed, and due to elevated kidney calcium clearance that exceeds the reabsorption ability, hypercalciuria occurs [17,24].

Calcium abnormalities in sarcoidosis (hypercalcemia and hypercalciuria) are responsible for further deterioration of kidney function during the disease course. On the one hand, hypercalcemia causes significant hemodynamic changes due to afferent vasoconstriction, leading to reduced glomerular filtration rate (GFR), elevated sodium excretion, together with polyuria, water depletion, and consequent AKI and tubular damage due to ischemia. On the other hand, sustained hypercalciuria leads to nephrolithiasis and nephrocalcinosis [17,25].

Similarly, in our case report, the patient presented with non-PTH-associated hypercalcemia, polyuria, metabolic alkalosis, hypercalciuria, and acute deterioration of kidney function. We initially assumed that the patient’s acute kidney failure and symptomatology occurred due to hypercalcemia and therefore the diagnostic algorithm primarily included other causes than non-PTH-related causes of hypercalcemia. Given that primary hyperparathyroidism, hyperthyroidism, malignancies, or drug-associated conditions were excluded, the suspicion of an underlying granulomatous disease was taken into consideration. The final diagnosis of sarcoidosis was made through a kidney biopsy, which eventually confirmed the disease.

Hence, the performance of renal biopsy in sarcoidosis has an exceptional role in the diagnosis of the disease, mainly in cases with isolated renal manifestations. The hallmark histological finding in RS remains the pattern of GIN, which is characterized by the presence of non-caseating epithelioid granulomas. In several studies, non-granulomatous interstitial nephritis is the most common histological pattern seen in approximately one half of cases, followed by GIN in 30%, IgA glomerulonephritis in 26%, and nephrocalcinosis in 11% of cases. However, a multicenter study including 47 patients with biopsy-proven RS revealed GIN as the most common histological pattern described in 66% of cases [23,25-27].

Histologically, GIN is considered a rather rare entity identified in approximately 0.5-0.9% of the performed kidney biopsies. The accurate incidence of GIN in sarcoidosis is not clearly defined, and its course is considered to be asymptomatic, while it rarely manifests as AKI or occurs in the absence of other extra-renal involvement. The above knowledge explains why autopsies in sarcoidosis patients reveal a higher incidence (~7-20%) of this histological finding [22,28-30].

Additionally, differential diagnosis in GIN consists of a wide range of conditions. The two most common etiologies are drug-induced and sarcoidosis, GIN representing up to 45% and 29% of cases, respectively. Infectious diseases, granulomatosis with polyangiitis (GPA), eosinophilic granulomatosis with polyangiitis, and tubulointerstitial nephritis with uveitis (TINU) are also reported as potential diagnostic alternatives. In our case, RS was suspected due to the patient’s initial clinical and laboratory findings, and the performance of renal biopsy revealed the presence of GIN. Taking into consideration that other causes of granulomatous nephritis, such as drugs, granulomatous infections, and GPA, were excluded, the diagnosis of sarcoidosis was confirmed [22,31-32].

Regarding the therapeutic landscape, data still remain ambiguous in sGIN [33]. Nevertheless, the importance of prompt onset of medication, especially in the setting of acute and threatening organ involvement, is considered imperative in order to avoid progression to chronic kidney injury or even to end-stage kidney disease (ESKD) requiring hemodialysis. Corticosteroids are the first-line therapy in sarcoidosis patients, and although steroid treatment is not well-standardized, in most cases, prednisone administration at a dose of 0.5-1 mg/kg per day is initiated in a gradually tapering scheme [9].

Several data suggest good responsiveness of GIN in steroid use with proven benefit in ameliorating kidney function decline and hypercalcemia, although in some cases, mild chronic kidney disease might develop. The extensive infiltration of giant cells, together with the presence of interstitial fibrosis in kidney biopsies, are the most vigorous factors for disease unresponsiveness and relapses to corticosteroid therapy. The antimalarial agent, hydroxychloroquine, is considered the second-line therapy for treating chronic hypercalcemia in sarcoidosis via reducing a1-hydroxylase hyperactivity by sarcoid granulomas, leading to reduced production of calcitriol and therefore decreased calcium intestinal absorption. Several data support the beneficial role of hydroxychloroquine as a steroid-sparing agent in RS, particularly in cases requiring long-term management to prevent relapses. Alternative immunosuppressant therapy options in patients with resistance, intolerance, or contraindications to steroid agents include azathioprine, methotrexate, mycophenolate mofetil, as well as tumor necrosis factor-alpha (TNF-α) inhibitors [33-38].

Finally, the results from the combined methylprednisolone (40 mg) and hydroxychloroquine (200mg) treatment demonstrated significant clinical improvement. Serum creatinine and calcium levels significantly improved within a few weeks of induction therapy, while follow-up imaging studies revealed important regression of lymphadenopathy. Moreover, on six-month follow-up, the patient demonstrated remarkable responsiveness to co-administration of corticosteroids with hydroxychloroquine, stabilization of renal function, and normal calcium levels.

## Conclusions

Sarcoidosis is a complex, multisystem disease with diverse organ involvement and nonspecific symptoms, making differential diagnosis challenging and increasing the risk of misdiagnosis. Renal involvement in sarcoidosis is reported infrequently and is associated with complex and interrelated underlying pathogenetic mechanisms, including mainly calcium-related abnormalities, GIN, glomerular diseases, and obstructive nephropathy. GIN is an uncommon histopathological pattern of renal injury presenting with or without other extra-renal manifestations, including constitutional symptoms. Early diagnosis and combined therapy with corticosteroids and hydroxychloroquine in cases with hypercalcemia seems to prevent progression of kidney injury and reduce morbidity and mortality.
